# Challenges to open innovation in traditional SMEs: an analysis of pre-competitive projects in university-industry-government collaboration

**DOI:** 10.1007/s11365-020-00727-1

**Published:** 2021-01-05

**Authors:** Alberto Bertello, Alberto Ferraris, Paola De Bernardi, Bernardo Bertoldi

**Affiliations:** 1 Department of Management, University of Turin, Turin, Italy; 2 Laboratory for International and Regional Economics, Graduate School of Economics and Management, Ural Federal University, Yekaterinburg, Russia

**Keywords:** Open innovation, Publicly funded projects, Small and medium-sized enterprises, University-industry-government collaboration, Challenges

## Abstract

Governments are increasingly focusing their efforts on stimulating innovation within small and medium-sized enterprises (SMEs). As a result, university-industry-government collaboration is gaining importance among the agenda of policymakers to enable open innovation in SMEs. However, these inter-organisational relationships often fail to meet expectations, especially when projects are oriented to pre-competitive R&D. Nevertheless, the literature has not yet provided sufficient evidence of the challenges related to the participation of traditional SMEs (i.e., low- and medium-low tech SMEs) in this specific type of collaboration. We collected qualitative data to analyse longitudinally three pre-competitive projects, exploring the main challenges faced by traditional SMEs. We have bracketed the projects in four phases: initiation and planning phase, execution phase, closing phase, and monitoring and control phase. For each of these phases we have individuated firm- and project-level challenges, providing practical and theoretical insights for open innovation scholars.

## Introduction

Our society is increasingly facing rapid and sudden changes caused by macro phenomena over which there is little control. The crisis triggered by the COVID-19 pandemic represents the latest emblematic example of how uncertain the economic scenario is and how companies must be able to develop increasingly immediate and disruptive responses to ensure competitiveness and sustainability. This is also affecting more traditional sectors and companies. In particular, many small- and medium-size enterprises (SMEs), which represent the core engine of the economy of many countries, seem to no longer have a secure future unless they constantly innovate their products, processes, and business models.

Recent literature on innovation and entrepreneurship has highlighted how the complexity of today’s business environment requires collaborative efforts by multiple actors (Bogers et al. 2018; Chesbrough and Bogers 2014; Enkel et al. 2020; Roundy 2017). This tendency is strongly eradicated in policymakers’ initiatives aimed at building formally structured clusters based on geographical proximity (Ferraris et al. 2020; Radziwon and Bogers 2019) to stimulate collaboration and higher innovation performance (Nestle et al. 2019; Parmentola et al. 2020). This of course also applies to low- and medium-low tech SMEs (hereafter, traditional SMEs) that are called to deal with the paradox of depending on external partners while having limited resources to manage such collaborative processes (De Bernardi et al. 2020; Santoro et al. 2018; Radziwon and Bogers 2019). Open innovation in traditional SMEs takes on different characteristics compared to open innovation in large companies and high-tech start-ups (Brunswicker and Vanhaverbeke 2015). Among the questions that arise, it is particularly relevant to investigate the challenges of the initiatives involving more traditional SMEs in open innovation. The open innovation body of literature has only recently focused on traditional SMEs (Dooley and O’Sullivan 2018; Martinez-Conesa et al. 2017), paving the way for addressing many interesting topics that deserve further attention. In particular, despite the increasing attention towards policies supporting collaborative innovation in SMEs (De Marco et al. 2020; European Commission 2020; Leckel et al. 2020), there is still a paucity of studies analysing formal pre-competitive collaboration between universities, industries, and governments. Pre-competitive open innovation refers to the exploration phase of R&D. In this case, SMEs tend to collaborate with research organisations and universities to develop basic research or research and experimental development. Projects can also be oriented to the commercialisation phase - also known as exploitation - of R&D. Although it is established among open innovation scholars that SMEs tend to prefer projects with a focus on the commercialisation phase of R&D (Spithoven et al. 2013; Verbano et al. 2015), the literature has not yet clarified the challenges related to the participation of traditional SMEs in basic research and research and experimental development. The need to address this lack of understanding has become even more vital now that policymakers are being called to boost the economy with targeted interventions to support SMEs to recover as soon as possible from the COVID-19 crisis (Organisation for Economic Cooperation and Development [OECD] 2020).

To shed further light on the challenges related to the participation of traditional SMEs in pre-competitive collaboration, our study analyses the case studies of three Italian regional projects based on university-industry-government collaboration. By conducting a participant observation, triangulated with interviews and document analysis, we followed a longitudinal perspective to investigate the main challenges at the firm- and project-level. We have found that: (1) the main challenges in the initiation and planning phase are lack of innovation strategy and a lack of partner mapping at the firm-level and goal incongruence and the presence of unknown partners at the project-level; (2) the major barriers in the execution phase of the project are inadequate information systems, time pressure, and lack of resources at the firm-level and goal redefinition, underestimation of partners’ efforts, and numerosity and heterogeneity at the project-level; (3) relevant challenges in the closing phase are lack of commitment post-project at the firm-level and delays in goal achievement and ineffective policies at the project-level; (4) finally, the most recurring barriers in the transversal phase of monitoring and control are inadequate management control systems at the firm-level and bureaucratisation at the project-level.

This study extends the literature on open innovation. In its early stages, research on open innovation has predominantly focused on the analysis of high-tech start-ups and incumbents while traditional SMEs, despite the importance occupied within national and regional economies, started attracting the attention only recently (Agostini and Nosella 2019; Radziwon and Bogers 2019; Vanhaverbeke et al. 2018). With this regard, we explicitly underline key challenges that typically occur in traditional SMEs in the pre-competitive phase, thus stimulating and improving the effectiveness of collaborative innovation. Moreover, by focusing on the challenges and performance drawbacks of the participation of SMEs in pre-competitive collaboration, we respond to the call for papers that explore the dark sides of open innovation (Stanko et al. 2017). A remarkable contribution is also provided by the nature of the study, which adopts a longitudinal perspective and a multi-level analysis to gain a thorough understanding of the complexity of open innovation (Bogers et al. 2017).

## Literature background

### Challenges to open innovation in SMEs

Most of the studies on open innovation and innovative collaboration have dealt mainly with large enterprises and start-ups active in R&D-intensive industries. Only recently this stream of literature has started opening up to SMEs to gain a deeper understanding on how the management and organisation of innovation is also developing in smaller and more traditional companies (De Marco et al. 2020; Dooley and O’Sullivan 2018). The first seminal work that analysed the challenges in SMEs adopting open innovation was carried out by van de Vrande et al. (2009). This study collected data from a large sample of Dutch companies and found that organisation and corporate culture-related issues that typically emerge when two or more companies are working together are clearly the most important barriers, followed by availability of time and resources and administrative burdens. All these barriers relate to specific typologies of open innovation. Organisational and cultural barriers are more prominent in venturing, participation in other firms, and involvement of external parties and users. Instead, availability of time and resources represent a barrier for almost all the open innovation practices investigated by van de Vrande et al. (2009), even when the intensity is not particularly high. Administrative burdens are again related to venturing, participation in other firms, and involvement of external parties, more specifically when cooperating with governmental or other not-for-profit institutions. Administrative burdens are also prominent when SMEs are provided with governmental subsidies and grants. The factors hindering the adoption of open innovation in SMEs have been also studied from Bigliardi and Galati (2016). Based on a survey of 157 Italian SMEs, they identified four main categories of barriers (i.e., knowledge, collaboration, organisational, and financial and strategic). As a result of a cluster analysis, Bigliardi and Galati (2016) found that the main barriers perceived by knowledge-intensive, high-tech and/or highly innovative industries are those related to the knowledge domain. The cluster of SMEs belonging to medium-innovative industries are mainly concerned with financial and strategic risks related to the implementation of the open innovation paradigm, as well as with knowledge barriers. Finally, the cluster composed by SMEs operating in traditionally less innovative sectors considers important collaboration and organisational barriers in adopting open innovation. Dufour and Son (2015) moved a step forward and after individuating four potential barriers in the literature, namely corporate culture, networking, organisational structure and knowledge management systems, they applied the theoretical framework to a single case study to provide insights on how to overcome these barriers by analysing the diverse organisational changes undertaken by the company. Ullrich and Vladova (2016) proposed a weighing and decision process framework as a conceivable solution approach to increment projects’ chances of success. According to their point of views, there are plenty of studies assessing the positive aspects of open innovation processes while few articles put emphasis on the dark sides. However, the best way to encourage open innovation in SMEs is to analyse the interdependencies of both facets and their combined impact on open innovation projects (e.g., Bresciani 2017).

### Pre-competitive collaboration and SMEs

The mainstream open innovation literature is still insufficient to provide a complete and detailed picture of the factors that hinder the adoption and the implementation of open innovation in SMEs. Van de Vrande et al. (2009) and Bigliardi and Galati (2016) have shown how specific open innovation practices and/or specific SME characteristics (e.g., size and sector) can generate different barriers, opening the way to more detailed reflections and in-depth case studies that focus on specific aspects related to the challenges SMEs undertake when engaging in open innovation, especially in inter-organisational projects (Santoro et al. 2019; Sultan 2020). Most of the time, inter-organisational projects are structured and coordinated by following logics that are more suitable for large companies than for SMEs, with the result that SMEs tend to occupy weaker network positions and to depend on partners’ strategies instead of having control on the direction of their open innovation efforts (Dodourova and Bevis 2014). The literature on open innovation has traditionally differentiated inter-organisational projects into two macro categories. The first category is the pre-competitive project and refers to the exploration phase of R&D. In this case, SMEs tend to collaborate with research organisations to develop basic research or research and experimental development. The second category refers to the commercialisation phase of R&D. In this case, companies usually play a more prominent role compared to research organisations. Less attention has been paid to SME involvement in horizontal collaboration, which is typically adopted by SMEs at the exploration stage of R&D collaborations (Lee et al. 2010; Narula 2004). Traditionally, SMEs tend to achieve better performance in terms of innovation outcomes when they engage in the commercialisation phase of a technology rather than its experimental and pre-competitive phase (Verbano et al. 2015). However, despite the difficulties in translating and exploiting research output successfully from research organisations in horizontal pre-competitive collaborations (Spithoven et al. 2013), SMEs will be increasingly called to engage in these partnerships to be competitive in an even more complex and uncertain environment where addressing and adjusting basic research to their needs can become a driver of sustainability in the long term.

### The problematic issue of innovation in traditional SMEs

With the term traditional SMEs, we refer to low- and medium-tech SMEs. According to the definition given by the OECD, the level of technology intensity in manufacturing companies can be categorised in low-tech, medium-low tech, medium-high tech, and high-tech. Following Hirsch-Kreinsen and Jacobson (2008), the categories that are not research intensive, namely the medium-low-tech and the low-tech, do not exceed the threshold of 3% of R&D intensity, while Som et al. (2015) categorise non-R&D-intensive companies as those whose R&D expenditure absorbed less than 2.5% of total sales. Collaborations between firms and research institutions tend to be privileged in industries with high technology intensity and rapid technological change, such as the pharmaceutical and engineering fields (D’Este and Patel 2007). This opens the door to the investigation on why and how lower-tech SMEs address open innovation and inter-organisational collaboration (Dooley and O’Sullivan 2018). Despite not being high-tech, SMEs often presents characteristics that are adapted to respond to changing contexts such as informal culture, flexibility and organic structure (Pullen et al. 2009; Giacosa et al. 2017). At the same time, however, they lack the resources to engage in university-industry-government collaboration that usually requires time to produce concrete results. On this basis, our study aims to further shed light on how traditional SMEs engage in open innovation by positing the following research question: “What are the main challenges that traditional SMEs face when participating in pre-competitive projects and how do these challenges unfold and influence each other along the phases of the project”?

## Methodology

### Research design

We have adopted an exploratory approach to investigate the challenges that traditional SMEs face in pre-competitive collaborative projects. A qualitative approach has been used to gain a more accurate picture of the dynamics of social relations that take place in inter-organisational projects, by examining the phenomenon of interest in its real-life context (Eisenhardt and Graebner 2007; Yin 2017). The choice of a multiple-case study as the design of this research allows us to follow a replication strategy that provides a strong basis for theory building (Eisenhardt and Graebner 2007; Yin 2017). By relying on the replication strategy, this study increases external validation of the findings, focusing the analysis on analytical rather than statistical generalisations (Yin 2017).

### Research context

We collected data from three pre-competitive projects based on university-industry-collaboration for basic research and experimental development. The cases belong to the Italian context. They have been selected after an online analysis of all the regional projects launched in Italy between 2010 and 2015. All three selected projects were part of a regional public tender launched in 2015 with a budget of €39,200,000 and had an estimated duration of 30 months, extended by six months in two cases due to slowdowns in achieving some of the priority objectives of the projects. These three projects were considered appropriate for our investigation because they met the following requirements: (1) the network was composed of at least one large company, one SME, and one research organisation or university; (2) at least 30% of the total eligible costs for the realisation of the project was allocated to SMEs; (3) taken together, these three cases covered a wide range of industries (e.g., car manufacturing, machine tools manufacturing, precision engineering, pharmaceutical, and agri-food sector). For the purpose of the study, we focused our analysis on traditional SMEs (i.e., low- and medium-low tech manufacturing SMEs with less than 3% of R&D intensity and 2.5% of total sales), which represent 55% of the SMEs involved in the three projects.

### Data collection

To deepen our understanding of the challenges that traditional SMEs face in pre-competitive university-industry-government collaboration, we developed a qualitative methodology able to produce in-depth and illustrative information on the various dimensions of the phenomenon under analysis, representing the views and perspectives of multiple actors (Eisenhardt and Graebner 2007; Yin 2017). For this reason, we used multiple sources of evidence (i.e., participant observation, interviews and document analysis) to triangulate data and to increase the richness of information (Yin 2017). First, we conducted a participant observation that consisted of participation in the steering committees, participation with the work packages meetings, management consultancy and visits to companies for field inspections. We also conducted informal interviews and took field notes during the events to analyse, in real time, feelings, reactions and meanings related to emerging issues.

In addition to the participant observation, we conducted 14 semi-structured interviews with SMEs’ managers, employees and other stakeholders of the project. We selected the key informants by following a purposive sampling technique and according to their knowledge and availability (Kumar et al. 1993). The sample was structured following the main aim of the study and the composition of the consortia (prevalence of SMEs). To ensure naturalness, interviewees were only made aware of the overall research purpose, without revealing specific questions, to prevent them from coming up with the answers in advance (Easton 2010). The interviews were conducted face-to-face or via skype by one researcher while the second researcher was in charge of taking field notes and scrutinising the behaviour and the approach of the interviewee in answering questions (De Bernardi et al. 2019). The same researcher was responsible for the transcription of the interviews. The protocol comprised a series of open-ended questions which were adapted according to the nature of the interviewee and the time frame. All of the interviewees were asked to explain motivations to join the project, expertise, initial goals and final outcomes, challenges in achieving the outcomes and possible solutions.

### Data analysis

**Fig. 1:**
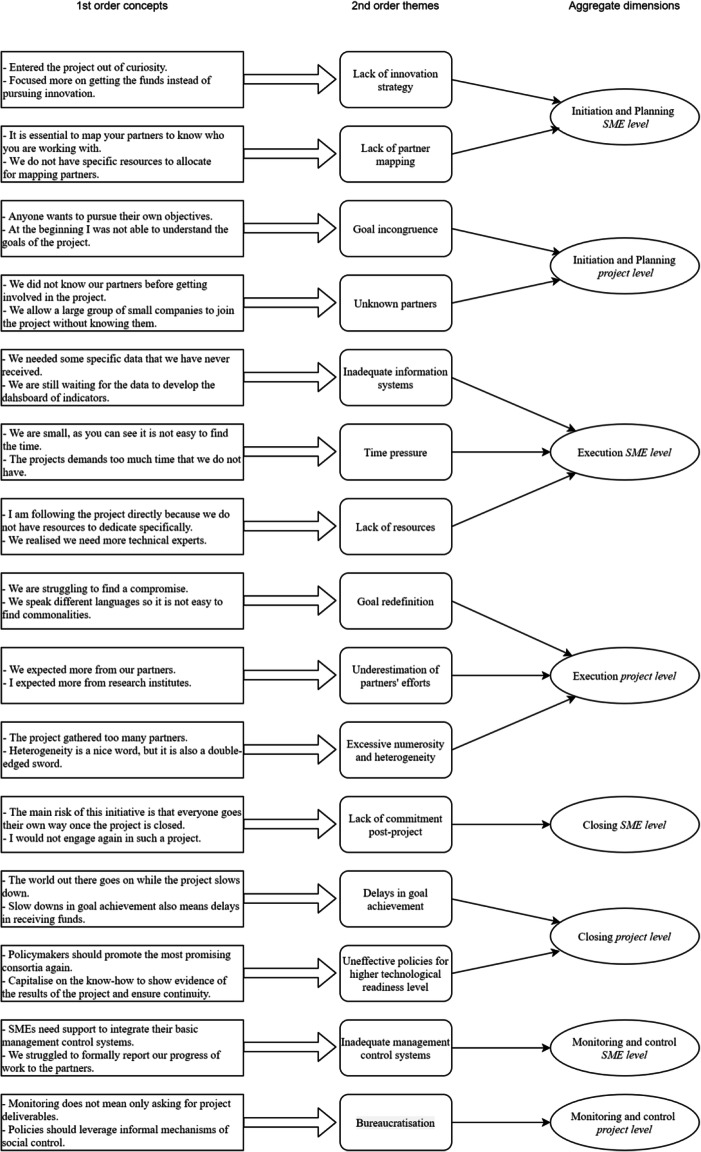
Data coding

Data were analysed by using the software NVivo, considering the field notes from observations, the transcribed interviews and the collected documents and texts as units of analysis (Eisenhardt 1989). We followed the Gioia methodology as “systematic approach to new concept development and grounded theory articulation that is designed to bring ‘qualitative rigor’ to the conduct and presentation of inductive research” (Gioia et al. 2013, p.15). In this phase, two authors generated the first order concepts by individuating any possible reference to the challenges hampering the effectiveness of open innovation in the SMEs participating in the three projects. Next, a process of seeking similarities and differences among the many categories was followed which resulted in the identification of second order themes. Once a workable set of themes and concepts was in hand and the theoretical saturation was reached (Glaser and Strauss 1967), we investigated whether it was possible to distil the emergent second order themes into our aggregate dimensions (Ferraris et al. 2019) resulting from the combination of temporal bracketing to analyse the data longitudinally (Langley 1999) and multi-level perspective to capture the complexity of open innovation (Bogers et al. 2017) (see Fig. 1).

## Findings

**Fig. 2:**
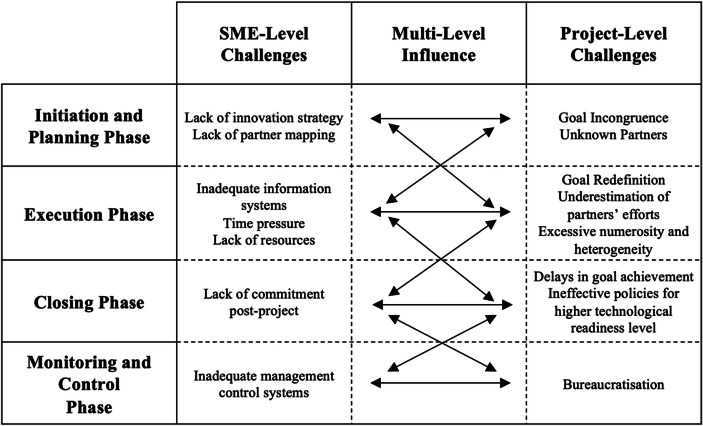
A multi-level analysis of the main challenges that traditional SMEs face in pre-competitive collaborative projects

In this section, we discuss the major findings of our study according to the four phases of the project: initiation and planning, execution, closing, and the transversal phase of monitoring and control (see Fig. 2).

### Initiation and planning phase

We have individuated several challenges during the planning phase which, despite its importance, is often overlooked by companies. These challenges often reveal an interplay between the SME- and project-level. For instance, most of the traditional SMEs in the projects did not seem to be too concerned about mapping the partners. Only a few companies spent time before the beginning of the project to learn about their partners. This caused problems during the definition of the goals of the project due to higher heterogeneity of partners combined with limited knowledge of each other as well as frictions and slowdowns during the execution of the project. Goal incongruence and the presence of unknown partners therefore depends not only on the design of the projects but also on the limited efforts of organisations to know their partners in advance. An SME manager justified himself by revealing that his company did not have enough time and resources to dedicate efforts to mapping the partners. He said, *“Of course it is important to know people in front of you well, especially because if you have to exchange information you have to trust them, but as a CEO of a small company we do not have specific resources to allocate for mapping partners”*. His statement highlights how the importance of knowing partners refers mainly to the social trust that can be created during the inter-organisational collaboration, while not considering the strategic implications of thoroughly knowing the other professional figures in the project. In fact, a deeper knowledge of a partner also enables synergies for the achievement of common goals according to the specific characteristics of the partners as well as preventing underestimating or overestimating partner efforts, which occurred during the execution phase. Another detrimental element was the lack of integration of the project in the organisational medium-long term vision. Some of the more structured companies took part in the projects with the aim of integrating the project itself within a precise strategic business plan. Other companies, on the other hand, did not have a precise research line and this made it difficult to plan the goals at the project-level and then to evaluate the final results. Many SMEs have attributed excessive importance to the minimisation of costs instead of co-creation of value with the other organisations. As a result, most of the funds allocated by the government have been used for ordinary activities instead of promoting innovation within the company.

### Execution phase

The goal inconsistencies were made explicit during the execution of the project. In these cases, many partners have decreased the intensity of collaboration while the frictions have increased. In an initial phase, an attempt was made to remedy this by redefining the objectives. However, this has not always led to the expected results. The difficulty in achieving the goals led to different scenarios. In a project, for instance, part of the consortium’s SMEs ran the risk of being relegated to a peripheral role and some of them even left the project or went bankrupt. In one project, the process of setting objectives underwent continuous redefinitions, causing slowdowns and inefficiencies. In this case, the project leader limited itself to making sure that each partner was able to achieve the minimum results required to receive the funds, with a negative impact in terms of joint value creation. As evidenced by the project manager of that project: *“Most of our end-users never provided the data in time and this made our job very difficult. This negatively affected all the modelling and the dashboard of indicators that we should have developed. [...] We had to find compromise solutions, considering that when a firm fails in its task, this negatively affected all the partners that are collaborating with that firm. In short, in the end we had to review most of the initial objectives, trying not to compromise anyone’s financing”*. Two projects also experienced problems due to the high number and heterogeneity of the partners. University-industry-government collaboration gathers actors with different values and cultures, and this makes the collaboration more complicated. A lack of attempts to learn about each other prior to collaborating has often resulted in underestimation of partner efforts. During the steering committees, for instance, when discussing the causes of slowdowns, many research organisations complained that SMEs were not providing the information necessary to go ahead with the project activities, while at the same time, SMEs were complaining that research organisations were not putting enough effort in their collaboration. The failure to provide information was found to depend mainly on two aspects. The first one is a motivational aspect. SMEs, and, more generally, the organisations involved in the project, tended to transfer only the strictly necessary information to protect their know-how as much as possible. The second one is a structural aspect. Traditional SMEs did not have the advanced information systems to obtain the information for which the research organisations and the other partners were asking.

### Monitoring and control phase

This phase intersects all the other phases of the project. In the cases under analysis, both the government and the project managers enabled mechanisms of monitoring and control to the work progress to ensure organisational compliance of all the organisations to the planned tasks. In every project, but even more where public funding is at stake, there is the need for strong regulation and monitoring. However, this resulted in an excessive number of tasks and deliverables, requiring SMEs to expend great efforts to provide the documentation to report the activities. Most traditional SMEs complained about *“the greatest difficulties in managing the administrative/bureaucratic part of the project”*. More specifically, they experienced an excess of documentation as well as an excessive bureaucratic burden of the platform on which they had to upload the documentation. Some tasks were perceived as too complicated, especially for those companies that have simplified accounting systems and limited human resources to devote to the project. A reduction in administrative burdens could free up resources for SMEs but should be accompanied by timely controls in the field to prevent companies from adopting a facade behaviour and obtaining the financing without having actually implemented their processes and innovated within their company. A project manager suggested that *“it is necessary to have more stringent monitoring of the funding body which does not mean only asking for project deliverables. [...] The officer carried out a couple of checks and mostly focused on analysing the documentation produced by the various partners, but visits to the company and participation in the meetings were particularly absent. In projects that involve such large monetary investments it is absolutely essential to have a tighter control that does not allow wasting money and that further allows the smallest companies to become aware that the financing and the relative investments can be virtuous and systematic”*.

### Closing phase

Two out of the three projects were postponed for six months due to the delay in reaching the objectives in some work packages. There were multiple causes and they depend on the critical issues analysed previously. The extension of the projects and the delays in reaching the objectives represented a deterrent to the desire of taking part in similar projects again. Many SMEs have in fact complained about the excessive slowness of the project activities (S12: *“The project started in 2015...right? And it will end in 2019, quite a long time for a research project of a small company. [...]It took two and a half years to put the first QR code on a product. The world out there goes on while the project slows down”*). Since inter-organisational projects are based on temporary collaborations, there is the concrete risk that at the end of the project all the partners return to their daily needs and activities, leaving the perspective of collaborating with each other. This is particularly frequent in SMEs, which have to deal with their day to day operativity. This practical conclusion is rather common, and it is precisely for this reason that policymakers, in this case regional governments, should look at it more carefully. According to the opinion of a project leader, *“the main risk that usually characterises 90% of these types of initiatives, is that everyone goes their own way once the project is closed. Except for a few virtuous relationships, which are established and develop mainly at the dyadic level, the network that has been created and that has given value to this project dissolves after the closure. This is quite natural because the network is composed by such different entities with different objectives, and when there is no longer a common goal the synergies that had been created get lost. [...] Policymakers should pay more attention to this element by promoting and financing the most promising consortia again, since they will presumably also create value in the subsequent projects”*.

## Discussion

### Theoretical implications

The main contributions of this study are specific to open innovation (and its subsets of university-industry-government relationships and publicly funded pre-competitive collaboration). Research on open innovation has traditionally focused on the analysis of large companies and high-tech start-ups (Brunswicker and Vanhaverbeke 2015), while only recently the attention has shifted to traditional SMEs, (Dooley and O’Sullivan 2018; Radziwon and Bogers 2019), and to the effective tools to promote inter-organisational collaboration (De Marco et al. 2020).

In order to provide a strong theoretical contribution to these research domains we analysed the challenges that can hamper effective collaborative innovation in SMEs as well as their effective involvement in regional entrepreneurial ecosystems (Brunswicker and Chesbrough 2018; Chesbrough and Brunswicker 2014; Laursen and Salter 2006; Radziwon and Bogers 2019; Zardini et al. 2018). SMEs are in fact traditionally oriented to engage in collaboration in the commercialisation phase of a technology rather than its experimental and pre-competitive phase (Verbano et al. 2015), such as the project we analysed. In this case, the presence of public incentives can be a breeding ground for open innovation and entrepreneurship, but it can also open the path to fraudulent and opportunistic behaviour (Wang et al. 2020). This study also follows Bogers et al.’s (2017) call for analysing open innovation at multiple levels. In fact, we developed an in-depth multi-level analysis to learn how organisational- and project-level challenges often influence each other. This analysis shows its usefulness in providing evidence of the multi-level nature of collaborative innovation as well as insights to the policymakers that will be further analysed in the next section. Moreover, since open innovation represents a broad domain, we analysed publicly funded collaborative innovation due to the increasing relevance that innovation policies are assuming for policymakers (De Marco et al. 2020; Leckel et al. 2020) and the lack of empirical evidence in the academic literature (Radziwon and Bogers 2019). More specifically, previous research on public incentives for spurring innovation and entrepreneurship among SMEs has focused on the impact of public subsidies on encouraging persistent R&D investment (Caloffi et al. 2018), the development of specific policies to stimulate effective innovation (De Marco et al. 2020; Leckel et al. 2020), and the comparison between collaborative and individual place-based programmes for SMEs (Bellucci et al. 2019). We add to this stream of literature by analysing publicly funded projects and the main challenges that characterise these initiatives.

### Practical implications

Public incentives for R&D are as diffused as they are questioned, since there is often contrasting evidence of their effectiveness. Indeed, publicly funded projects can be a powerful tool for enhancing collaborative innovation in less experienced SMEs, but, at the same time, SMEs can use public money for ordinary activities, minimising costs instead of effectively innovating (Wang et al. 2020). The challenges individuated in this study suggest some possible responses at the project-level to improve the positive impact of these initiatives.

To reduce the effort of SMEs, projects should activate mechanisms, such as internships, to provide SMEs with human resources to dedicate to the project. Eventually, incentives could be allocated to also encourage the hiring of these resources within companies and ensure greater continuity. In this regard, it would also be interesting if the funding body of the initiative can in some way guarantee an engagement in a subsequent project oriented towards the R&D commercialisation phase for those consortia that have proven to be productive and successful.

Regarding the accounting aspect of the projects, considering that SMEs often have basic accounting systems and that relying on external consultants could represent an excessively expensive and detrimental cost for SMEs, the projects should consider creating a special body that can support SMEs with coaching activities for project reporting.

Then, since collaboration among different stakeholders often leads to frictions and misunderstandings, it is important to pay particular attention to the selection phase of the project, which has not yet been deeply investigated by the literature. To reduce opportunistic behaviour, the lack of clear ideas already mentioned and the lack of prior mutual knowledge, the projects should first consider reducing the numerosity and the heterogeneity of the consortia, and, second, they should establish clear and rigorous criteria for the process of consortium creation. This means that the project leader should directly and personally select the SMEs whose goals are supposed to be aligned with the scope of the project. The underestimation of partners’ efforts should be reduced by incentivising mutual understanding. In this regard, it is important that projects leverage mechanisms that increase trust between partners. When there are differences in terms of language, culture and organisational structure, it is important to stimulate informal knowledge exchange to encourage interactions that are not limited to the official steering committee or to the formal deliverables. Visits to the companies and informal sessions during the steering committees could leverage informal mechanisms of knowledge exchange and social control to increase mutual understanding within the project.

### Limitations and future development

Despite the novel insights gained from this research, the study is not free of limitations. First, even if the qualitative methodology extends the previous knowledge on the dynamics on traditional SME engagement in collaborative innovation, a mixed method could further enrich the results by combining analytical and statistical approaches. Second, this study developed its analysis over time along the whole duration of the projects, from the beginning to the end. However, it would be interesting to monitor SMEs over time after the end of the project as well. For example, SMEs should be stimulated and incentivised to participate in smart city projects where a city’s ecosystems of partners and the collaborations between them are critical for innovation and where entrepreneurships and project level barriers are common (Scuotto et al. 2016; Sandulli et al. 2017; Bresciani et al. 2018). Future studies should address this gap by further extending the longitudinal perspective.
